# Nutritional and Techno-Functional Properties of Ultrasound-Assisted *Moringa oleifera* Leaf Protein Concentrate with Potential Applications in Food Gels

**DOI:** 10.3390/gels11110843

**Published:** 2025-10-22

**Authors:** Eunice Tranquilino-Rodríguez, Estefanía Bautista-Durán, José Juan Virgen-Ortiz, Ma. Guadalupe Garnica-Romo, Osvaldo Alvarez-Cortés, Gabriela Monserrat Ochoa-Manzo, Héctor Eduardo Martínez-Flores

**Affiliations:** 1 Facultad de Químico Farmacobiología, Universidad Michoacana de San Nicolás de Hidalgo, Morelia 58240, Michoacán, Mexico; eunice.tranquilino@umich.mx (E.T.-R.); 1647719e@umich.mx (E.B.-D.); osvaldo.alvarez@umich.mx (O.A.-C.); 1243773e@umich.mx (G.M.O.-M.); 2 SECIHTI–Centro de Investigación en Alimentación y Desarrollo, A. C. (CIAD)–CIDAM, Antigua Carretera a Pátzcuaro Km 8, Morelia 58341, Michoacán, Mexico; jose.virgen@ciad.mx; 3 Facultad de Ingeniería Civil, Universidad Michoacana de San Nicolás de Hidalgo, Santiago Tapia 403, Col. Centro, Morelia 58000, Michoacán, Mexico

**Keywords:** *Moringa oleifera*, protein concentrate, ultrasound, amino acids, gelation

## Abstract

*Moringa oleifera* leaves are a protein-rich source containing all essential amino acids and offering high nutritional value. Ultrasound-assisted extraction (UAE) has emerged as an efficient method to improve protein recovery while enhancing the structural and functional properties of plant proteins. This study aimed to improve protein extraction from *M. oleifera* leaves using UAE and to characterize the nutritional composition and gel-related properties of the resulting protein concentrate. Chosen conditions were a solubilization pH of 11.68, 20 min of ultrasound treatment, and precipitation at pH 4.5, resulting in an extraction yield of 79.90% and protein content of 53.97%. *Moringa oleifera* leaf flour (MOF) contained 29.38% protein, 37.98% dietary fiber, and high mineral levels (1751.85 mg/100 g of calcium; 512.55 mg/100 g of magnesium). Compared with MOF, the *M. oleifera* protein concentrate (MOPC) showed a 21.4% increase in essential amino acids, with leucine and lysine being the most abundant. Functionally, MOPC exhibited 24.26% solubility at pH 2, complete gelation at pH 8, 58.66% emulsifying capacity with 79.52% stability at pH 10, and 21.11% foaming capacity with 94.44% stability at pH 2. The gel-forming ability was the most promising characteristic, highlighting the potential of MOPC as a natural structuring agent in gel-based food systems and functional formulations.

## 1. Introduction

Protein–energy malnutrition remains a major global public health challenge, especially in developing and low-income countries. The World Health Organization (WHO) reports that, in 2024, an estimated 150.2 million children under 5 years of age were stunted, 42.8 million were wasted, and 35.5 million were overweight—figures that reflect both inadequate protein and energy intake as well as broader nutritional imbalances [1]. Proteins are essential for growth, reproduction, and recovery from disease; however, the rising demand for animal-derived protein is associated with adverse health outcomes and substantial environmental impacts [2]. In this context, plant-based proteins present a sustainable and cost-effective alternative, with growing evidence supporting their role in metabolic and cardiovascular health [3,4].

*Moringa oleifera*, a member of the *Moringaceae* family native to northwestern India and now cultivated worldwide [5], has gained recognition as a nutrient-dense plant. Its leaves contain 20–35% protein, all essential amino acids, vitamins, minerals, fatty acids, and phenolic compounds [6,7]. Owing to this comprehensive nutritional profile, *M. oleifera* leaves are considered a valuable protein supplement for food products, with the potential to mitigate malnutrition and support health in vulnerable populations [8]. Beyond their nutritional content, *M. oleifera* proteins also exhibit promising functional properties—particularly emulsification, foaming, and gelation—which are crucial for designing novel food matrices and gel-based delivery systems.

Several technologies have been developed to improve protein extraction from plant matrices, spanning from conventional chemical methods to advanced green approaches such as enzyme-assisted extraction, ultrasound, pulsed electric fields, microwaves, high pressure, and hybrid strategies that combine these techniques [9,10]. While some studies report higher extraction yields from traditional protein alkaline solubilization, others highlight ultrasound-assisted extraction (UAE) as especially effective, largely due to acoustic cavitation that enhances cell disruption and mass transfer [11,12]. These diverging findings underscore the need to refine hybrid methods that balance yield with preservation of protein functionality.

Protein techno-functional properties—such as solubility, emulsifying and foaming capacity, and especially gel-forming ability—are strongly shaped by extraction technique. These characteristics determine the capacity of proteins to create structured networks that stabilize emulsions, retain water, and form gel matrices with desirable textural and mechanical attributes. In conventional and ultrasound-assisted methods, protein extraction is often combined with alkaline solutions with pH values above 8.0 [13]. In the case of wheat germ, the application of ultrasound increased protein extraction yield by between 37% and 57% [14]. Similarly, during the optimization of protein extraction from rice bran using an ultrasonic bath, an average yield of around 15% was achieved [15].

The type of matrix used in extraction also has a significant influence on process performance. It has been shown that reducing particle size improves extraction efficiency by increasing the contact surface between the solvent and the sample. In a previous study, the effectiveness of ultrasound-assisted extraction (UAE) for protein recovery from sunflower meal (*Helianthus annuus* L.) was evaluated. The application of ultrasound at different frequencies (20–50 kHz) with a power of 220 W/L for 15 min in an alkaline aqueous medium (pH 8.0) produced a significant increase in process efficiency, raising the protein extraction yield from 28.0% to 54.26% [16]. Recent studies suggest that combining different extraction techniques can further enhance these properties [17,18]. The identification of suitable process parameters is critical for producing functional protein concentrates suitable for gel-based foods and structured matrices.

Therefore, this study, for the first time, we propose an integrated approach that combines protein extraction from *M. oleifera* leaves using alkaline solubilization whit UAE and isoelectric precipitation. The nutritional composition and functional properties of the resulting protein concentrate were characterized, with particular focus on its gel-forming capacity. The findings highlight its potential as a sustainable structuring agent for innovative gel-based food systems.

## 2. Results and Discussion

### 2.1. Optimization of Protein Extraction from M. oleifera Leaves

Table 1 presents the results of the central composite design (CCD) study, which evaluated the effects of solubilization pH, ultrasound time, and precipitation pH on protein extraction yield and protein content. Among the treatments, Treatment 5 produced the best results, with a solubilization pH of 11.68, 20 min of ultrasound treatment, and a precipitation pH of 4.5. Under these conditions, the extraction yield reached 82.44%, with a protein content of 50.39%.

**Figure 1:**
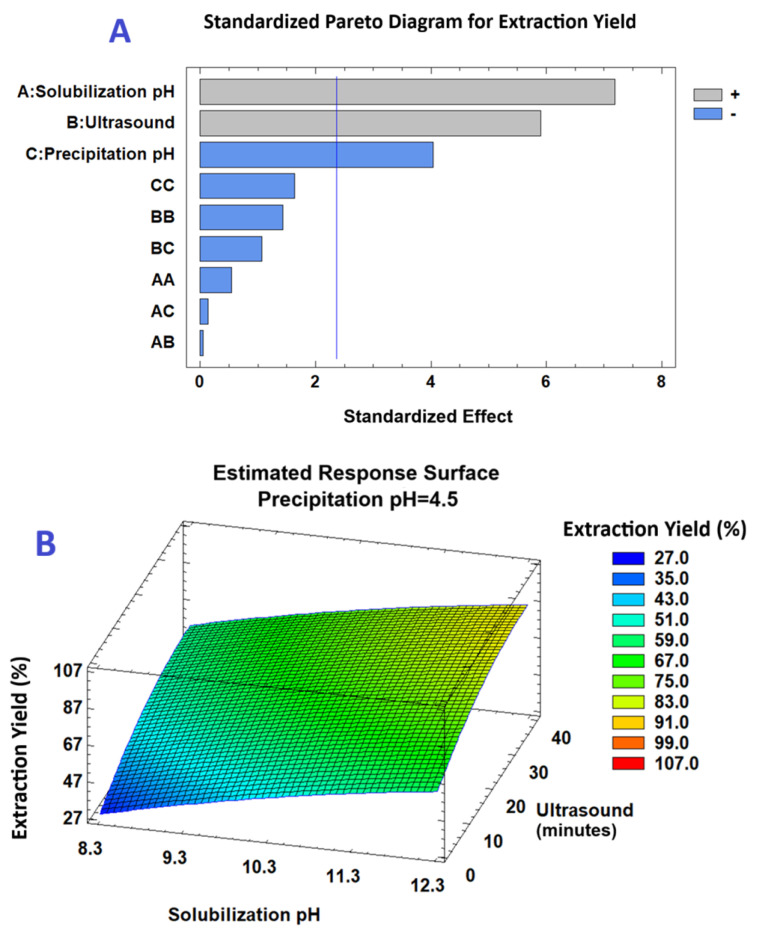
(**A**) Pareto plot (*p* = 0.05) showing the standardized effects of factors on extraction yield from *M. oleifera* leaves. (**B**) Three-dimensional response surface illustrating the combined effects of solubilization pH and ultrasound time on extraction yield (%) at a constant precipitation pH of 4.5.

Figure 1A (Pareto diagram) and Table 2 (ANOVA) show that solubilization pH and ultrasound time had significant positive linear effects on extraction yield (*p* ≤ 0.05), while precipitation pH showed a significant negative linear effect (*p* ≤ 0.05). Quadratic effects and interactions were not significant. A positive linear effect of solubilization pH indicates that maximum protein solubility is achieved under more alkaline conditions, resulting in a higher extraction yield. Similarly, the positive linear effect of ultrasound time suggests that longer exposure times encourage the disruption of plant cell walls and subsequent release of proteins encapsulated within the cell matrix. This increases the efficiency of the extraction process. A negative linear effect of precipitation pH indicates that the isoelectric point (pI) of proteins is lower. This shows that the extraction yield increases as the precipitation pH decreases. The three-dimensional response surface in Figure 1B further illustrates that the highest yield was obtained at higher solubilization pH and longer ultrasound times, with precipitation pH held constant at 4.5.

**Figure 2:**
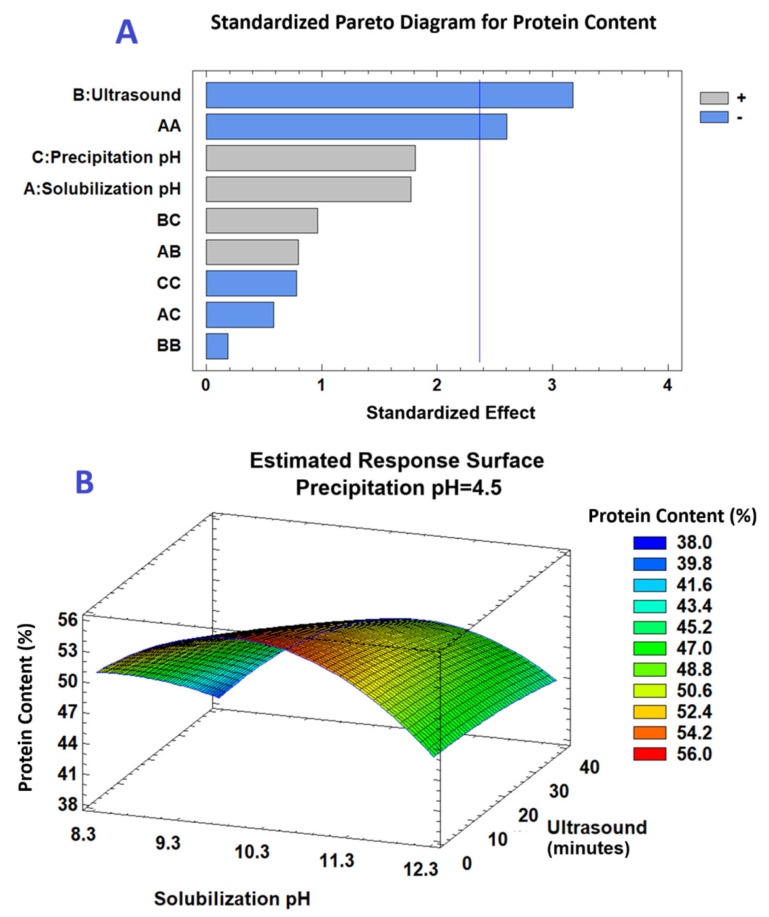
(**A**) Pareto plot (*p* = 0.05) showing the standardized effects of extraction factors on protein content from *M. oleifera* leaves. (**B**) Three-dimensional response surface illustrating the effects of solubilization pH and ultrasound time on protein content (%) at a constant precipitation pH of 4.5.

Figure 2A present the Pareto diagram and Table 3 (ANOVA) for protein content, which shows a significant negative linear effect of sonication time and a significant negative quadratic effect of solubilization pH (*p* ≤ 0.05). The response surface in Figure 2B indicates that maximum protein content was obtained at an intermediate solubilization pH and low sonication time, with precipitation pH fixed at 4.5.

Equations (1) and (2) represent the fitted models predicting extraction yield and protein content, respectively, based on the independent variables in their original units. The models showed strong agreement between experimental and predicted values, with R^2^ = 0.93 for extraction yield and R^2^ = 0.78 for protein content.

(1) $$\begin{array}{ll} Extraction\;yield\; & \left(\%\right)\\ & =-153.108+23.04\times A+2.20208\times B+19.2465\times C\\ & -0.684964\times A^{2}-0.00668375\times \left(A\times B\right)-0.201087\times \left(A\times C\right)\\ & -0.0182137\times B^{2}-0.161119\times \left(B\times C\right)-2.07565\times C^{2} \end{array}$$

(2) $$\begin{array}{ll} Protein\;content\; & \left(\%\right)\\ & =-143.952+36.2995\times A-1.10663\times B+8.78229\times C\\ & -1.72142\times A^{2}+0.0624013\times \left(A\times B\right)-0.456762\times \left(A\times C\right)\\ & -0.00122942\times B^{2}+0.0757163\times \left(B\times C\right)-0.515635\times C^{2} \end{array}$$

Optimization of the individual responses predicted stationary or maximum values of 90.48% for extraction yield (solubilization pH: 11.67; ultrasound: 36.81 min; precipitation pH: 2.81) and 54.98% for protein content (solubilization pH: 10.02; ultrasound: 3.18 min; precipitation pH: 4.30). Simultaneous optimization produced a desirability of 0.72, with optimal conditions at a solubilization pH of 10.95, 23.47 min of ultrasound, and a precipitation pH of 4.24, to obtain an extraction yield of 75.92% and a protein content of 50.80%. However, experimental Treatment 5 of the CCD (solubilization at pH 11.68 for 20 min and precipitation at pH 4.5) proved more effective, with an extraction yield of 82.44% and protein content of 50.39%.

Treatment 5 was validated in triplicate, yielding an extraction yield of 79.90% ± 2.53% and a protein content of 53.97% ± 1.43%. These values fall within the expected range of the optimization design, with deviations of 3.58% for yield and 2.54% for protein content.

Although the model predicted a point of maximum desirability, experimental validation showed that one of the conditions in the matrix (Treatment 5) offered superior and reproducible results. Therefore, we prioritized reporting this condition as optimal in practice, which does not invalidate the optimization but rather complements it with experimental verification to ensure that the proposed conditions are efficient and reproducible.

Conventional protein extraction methods often require long processing times and the use of solvents or extreme pH and temperature conditions, which can limit both yield and the functional properties of extracted proteins [19]. In this study, isoelectric solubilization and precipitation were combined with UAE to improve protein yield and content from *M. oleifera* leaves. UAE has emerged as an efficient and promising technology for protein recovery due to its multiple advantages, including selectivity, cost-effectiveness, and a significant reduction in both processing time and solvent usage. A key aspect of this technique is its operation at low temperatures, which minimizes thermal denaturation of proteins and, consequently, preserves or even enhances their functionality. This benefit has been highlighted in several studies on the application of emerging technologies for protein extraction from legumes [20]. UAE offers advantages such as reduced processing time and minimized thermal denaturation. However, key parameters—particularly pH and sonication time—must be carefully selected because excessive ultrasound exposure can lead to protein aggregation, hydrolysis, or denaturation [21,22].

As shown in Table 1, Treatment 5 employed a solubilization pH of 11.68, which promoted amino acid ionization and disulfide bond cleavage, thereby enhancing protein solubility and extraction [23]. In this study, a sonication time of 20 min provided the best results; shorter treatments have been reported to decrease recovery, while longer exposure may induce denaturation and aggregation due to hydroxyl radical formation [10]. Thus, selecting an appropriate sonication time was essential. The precipitation pH of 4.5 identified in this study aligns with the reported isoelectric point of *M. oleifera* leaf proteins (pH 4–4.5) [24]. At this pH, the reduced net protein charge lowers solubility, facilitating efficient precipitation.

Response surface methodology (RSM) is a powerful multivariate statistical tool that enables optimization of extraction processes while reducing the number of experimental runs and conserving time and resources [25]. In this study, application of a CCD within RSM yielded a protein extraction yield of 79.90% ± 2.53% from *M. oleifera* leaves. This extraction yield exceeds previously reported yields for seeds of the same species (39.9%) [26] and for leaves (28.93%) [12], both obtained using RSM combined with UAE. The high yield observed here was attributed to ultrasound treatment, in which cavitation and shear forces disrupt cell walls, increase solvent contact surface area, and accelerate mass transfer, thereby enhancing protein recovery [27]. In this study, the best conditions of solubilization pH, ultrasound time, and precipitation pH were identified to obtain a protein concentrate with 53.97% ± 1.43% protein. According to the established classification by the Food and Agriculture Organization of the United Nations [28], this corresponds to a concentrate (50–80% protein). A comparative study was conducted to evaluate the efficiency of enzyme-assisted extraction (EAE), ultrasound-assisted extraction (UAE), and microwave-assisted extraction (MAE) for obtaining protein isolate from *Moringa oleifera* seeds in comparison with conventional alkaline extraction (CE). The results indicated that all assisted techniques generated high-purity isolates (80.57–86.61 g/100 g). The UAE sample had the highest extraction yield and the best thermal stability. The MAE sample had the highest protein content, greater surface hydrophobicity, and better emulsifying capacity, but slightly lower solubility. Taken together, these findings demonstrate that emerging technologies, especially UAE and MAE, outperform the conventional method by differentially optimizing protein extraction yield and protein purity [29].

### 2.2. Nutritional Profile of M. oleifera Flour and Protein Concentrate

Table 4 presents the proximate composition of *M. oleifera* flour (MOF) and *M. oleifera* protein concentrate (MOPC). The moisture contents were 7.71% and 5.09%, respectively, values comparable to the 7.23% reported in the literature for *M. oleifera* leaves [30]. The protein content increased from 29.38% in MOF to 53.97% in MOPC, representing an 83% enrichment. This level is higher than the 45.5% protein yield previously reported from *M. oleifera* leaves extracted with UAE [31]. The substantial increase in protein concentration underscores the potential of MOPC as a nutritional supplement for preventing and addressing malnutrition in vulnerable populations, including children and the elderly of developing countries [32].

Lipid content increased from 5.46% in MOF to 15.35% in MOPC, likely due to ultrasonic cavitation, which facilitates the release of proteins and lipid compounds such as fatty acids, phytosterols, and fat-soluble pigments [33]. By contrast, carbohydrate content decreased from 10.44% in MOF to 5.43% in MOPC, while dietary fiber decreased from 37.98% to 18.09%, reflecting the enrichment of protein. Despite this reduction, MOPC retains a moderate fiber content, which has been associated with hypoglycemic effects and improved digestion [30].

The ash content of MOF was 9.03%, consistent with previously reported values (8.05–10.38%), reflecting its high mineral composition [34,35]. In MOPC, mineral content decreased to 2.07% as a result of processing. Given the higher mineral content in MOF, key minerals of nutritional and functional importance—including calcium, magnesium, potassium, iron, and sodium—were quantified individually [36].

As shown in Table 5, MOF is a rich source of calcium (1751.85 mg/100 g), magnesium (512.55 mg/100 g), potassium (1144.58 mg/100 g), and sodium (85.81 mg/100 g), with values generally within the ranges reported in the literature [37]. The calcium and magnesium contents observed here exceeded those previously reported [30], namely 1480 mg/100 g and 301.11 mg/100 g, respectively. Variations in the nutritional composition of MOF, including both macro- and micronutrients, are influenced by factors such as climate, soil type, crop management, cultivation status (wild or cultivated), plant maturity at harvest, and post-harvest processing methods [38]. Overall, the nutritional values obtained in this study were comparable to, or in some cases higher than, those reported previously.

Compared with the daily recommended intakes established by the Pan American Health Organization [39], MOF provides sufficient calcium and magnesium to meet the needs of both children and adults, supporting bone health and metabolic regulation. Although phosphorus, iron, and sodium are present at lower concentrations, they may still contribute to bone development, hemoglobin synthesis, and electrolyte balance. Overall, MOF demonstrates a valuable mineral profile for human nutrition [40,41].

### 2.3. Amino Acid Profile

Table 6 presents the most recent amino acid requirement values established by the Food and Agriculture Organization [42], which represent the reference amino acid pattern to which a high-quality protein should conform. A protein source that provides at least these amounts of each essential amino acid per gram of protein is considered to meet the requirements for adults [42]. Table 6 also shows that 17 amino acids were identified in MOF and MOPC, including all essential amino acids. In MOF, leucine was the only limiting amino acid, satisfying 82.3% of the FAO and IAEA reference requirements [42]. In MOPC, the limiting amino acids were methionine and cysteine, which together supplied 60.4% of the recommended levels, according to the FAO and IAEA. The most abundant essential amino acid in MOF was lysine (102.13 mg/g), while glutamic acid (229.01 mg/g) was the predominant non-essential amino acid. In MOPC, leucine (110.98 mg/g) and glutamic acid (136.39 mg/g) were the most abundant essential and non-essential amino acids, respectively. Previous studies have reported histidine and methionine as the limiting amino acids in *M. oleifera* leaves [43]; however, in the present study, histidine concentrations were found to be adequate. Because plant proteins often have incomplete amino acid profiles, the food industry frequently relies on complementary proteins or exogenous amino acid supplementation. In this context, MOPC represents a valuable source of essential amino acids, supporting protein synthesis, bone health, and immune and neurological function [44,45,46,47].

These findings are consistent with a previous study reporting a 21.2% enhancement in essential amino acids following enzymatic protein extraction from *M. oleifera* leaves, where values rose from 402.9 mg/g in the leaves to 488.6 mg/g in the protein concentrate [48].

Processing *M. oleifera* leaves into flour has also been shown to enhance nutritional value by improving nutrient bioavailability [49]. In one study, children with severe acute malnutrition who received daily doses of moringa leaf powder for 2 months demonstrated weight gain and reduced severe wasting compared with those on a standard diet [50].

UAE has been reported to improve both the amino acid profile and digestibility of plant proteins by increasing their accessibility to digestive enzymes [44]. In this study, MOPC contained nearly all essential amino acids at levels exceeding FAO recommendations [51], underscoring its potential as a plant-based protein source for functional foods and nutraceuticals that support health and help prevent metabolic and degenerative diseases.

### 2.4. Physicochemical and Functional Properties of MOPC: Potential for Gel Formation

As shown in Table 7, MOPC had a pH of 4.63, resulting from isoelectric precipitation, and a water activity of 0.44—conditions that favor powder stability by slowing enzymatic reactions and inhibiting microbial growth [52]. The powder exhibited a dark green, opaque color (L* = 23.90, C* = 16.36, h° = 88.11), consistent with the oxidation of phenolic compounds during alkaline extraction, as previously reported [53].

Water and oil retention capacities were 1.66 g/g and 2.52 g/g, respectively, exceeding the 1.12 g/g and 1.78 g/g reported for *M. oleifera* leaf protein extracted by ultrasound [12].

Previous studies have demonstrated that the susceptibility of proteins to ultrasound treatment depends primarily on their native conformation rather than their biological origin, which consequently influences improvements in their functional properties. Proteins with less ordered structures, such as caseinate (CAS), exhibited greater sensitivity to ultrasonic disruption, as evidenced by pronounced alterations in secondary structure—specifically, a 26.1% reduction in α-helix content and a 39.4% increase in random coil formation. Conversely, the effect of ultrasound was more limited in compact globular proteins, such as chickpea protein isolate (CPI), likely due to their smaller particle size (108 nm). These conformational modifications were directly correlated with enhanced functional performance, notably improving the physicochemical stability of CAS-stabilized oil-in-water (O/W) emulsions. Such improvements are attributed to ultrasound-induced structural rearrangements that generate a more porous protein network and expose hydrophobic groups, thereby enhancing the protein’s affinity for both aqueous and lipid phases [54]. Therefore, these improvements in MOPC are attributed to ultrasound-induced structural modifications, which create a spongier protein matrix and expose hydrophobic groups, thereby enhancing water and oil affinity. The presence of hydrophilic fibers in the leaves also contributes to these functional properties [44,55,56]. Such hydration-related attributes are particularly relevant for gel-based systems, where water binding plays a key role in defining texture and network stability.

*Moringa oleifera* proteins exhibit multiple functional properties relevant to food development, including solubility, gelation, emulsification, and foaming capacity. Previous studies have shown that UAE can increase the solubility of *M. oleifera* seed and leaf proteins by up to 29.82% and 24.82%, respectively [12,26]. In the present study, the protein concentrate achieved a solubility of 24.26% at pH 2 (Table 7). Ultrasound-induced modifications likely exposed hydrophilic amino acid groups, thereby improving protein–water interactions [57]. Improved solubility under acidic conditions is highly desirable for protein dispersion prior to gel formation.

The gelling capacity of *M. oleifera* proteins is largely attributed to their high content of hydrophobic amino acids, which facilitate the development of three-dimensional protein networks [44]. Gel formation depends on the balance between hydrophobic and electrostatic interactions, which govern protein–protein and protein–solvent associations in gelling systems [58]. In this study, MOPC exhibited 100% gelation at pH 8, demonstrating that alkaline conditions enhance these interactions, consistent with findings reported in the literature [59]. This result surpasses previously reported values for isoelectric-precipitated isolates, which achieved only 78% gelation at pH 7 [60]. Another recent study that used alkaline extraction with isoelectric precipitation to isolate proteins from *Moringa oleifera* seeds reported limited gelation of 20% at pH 8 [61]. Thus, UAE not only improved yield but also significantly strengthened gel-forming ability, positioning MOPC as a promising candidate for gel-based functional foods and structuring agents. Its application could be extended to the formulation of gelled products, such as dairy desserts, fortified protein gels, fat substitutes, structured matrices for controlled nutrient release, and textured vegetable products. In addition, recent research supports the potential of *Moringa oleifera* protein in gel-type food systems. One study reported that the incorporation of amylose significantly improves the firmness, water retention capacity, and reticular structure of gel made from *M. oleifera* seed protein [62]. Similarly, it has been reported that the addition of this protein to rice starch gels reinforces the three-dimensional network, increases the rheological modules (G′ and G″), and improves gel strength [63]. Taken together, these results suggest that *M. oleifera* protein concentrate (MOPC) not only has remarkable gelling capacity but can also enhance the functionality of gel-type food matrices, expanding its application as a functional ingredient in the development of innovative foods.

The foaming capacity of MOPC reached maximum values of 21.11% at pH 2 and 20.00% at pH 10, with high stability (>90%) observed only under acidic conditions. These results are consistent with previously reported improvements in *M. oleifera* seed proteins following ultrasound treatment [26]. While foaming offers potential for beverage applications, it is also relevant to aerated gel systems, where foam stability contributes to novel textures.

Similarly, the emulsifying capacity (58.66%) and stability (79.52%) of MOPC at pH 10 are consistent with previous findings, which show that emulsification improves away from the isoelectric point. These values exceeded those reported for conventionally extracted isolates [60]. Ultrasound promoted protein disaggregation and structural reorganization, increasing the exposure of hydrophobic residues and thereby improving adsorption at the oil–water interface [12,64]. Enhanced emulsification is valuable not only for conventional foods (ice cream, processed meats, dressings) [65] but also for emulsion-filled gels, which integrate emulsification with gelation to create multifunctional delivery systems.

Collectively, these results demonstrate that UAE improved both the nutritional and functional properties of MOPC, with gelation standing out as the most promising characteristic for structuring protein-based food systems.

## 3. Conclusions

This study demonstrated that combining alkaline solubilization, ultrasound-assisted extraction, and isoelectric precipitation substantially increased extraction yield from *M. oleifera* leaves, achieving 79.90% with a protein content of 53.97% under enhanced conditions. *Moringa oleifera* leaves are naturally protein-rich, providing all essential amino acids along with high levels of calcium and magnesium. Concentration increased protein content. It also increased the levels of amino acids. These amino acids include leucine, phenylalanine, tyrosine, and tryptophan. In addition to nutritional enrichment, the resulting protein concentrate exhibits remarkable pH-dependent techno-functional properties, such as complete gelation capacity under alkaline conditions, as well as outstanding water retention, emulsion, and foaming capacity. These characteristics reflect a well-structured protein network, promoted by ultrasound-assisted extraction. Taken together, these results position MOPC as a multifunctional ingredient with great potential for the development of gel-structured systems, dairy desserts, fat substitutes, controlled-release matrices, and textured plant-based products. Therefore, *M. oleifera* represents a sustainable and versatile source of protein with promising applications in the formulation of functional and innovative foods that contribute to the fight against malnutrition and the emerging market for alternative proteins.

## 4. Materials and Methods

### 4.1. Biological Material

*Moringa oleifera* leaves were collected in Salguero, Michoacán, Mexico. Taxonomic identification and classification were performed by the Herbarium of the Faculty of Biology (EBUM), Michoacán University of San Nicolás de Hidalgo, where a voucher specimen was deposited (folio no. 18610).

### 4.2. Production of MOF

*Moringa oleifera* leaves were disinfected with 70% ethanol for 5 min, shade-dried for 72 h, and subsequently crushed and sieved to obtain flour with a particle size of <250 μm. The flour was stored at 4 °C until further analysis.

### 4.3. Optimization of Protein Extraction from M. oleifera Leaves

Protein extraction from MOF was performed using a CCD within the framework of RSM. The experimental factors evaluated were solubilization pH (9, 10, and 11), ultrasound treatment time (10, 20, and 30 min), and precipitation pH (3.5, 4.5, and 5.5). These ranges were selected based on previous studies conducted under similar conditions [26,60], as well as preliminary internal testing which confirmed that protein recovery decreases outside of these ranges.

As shown in Table 8, the CCD under the RSM framework included 17 experiments, with 3 replicates at the center point and 2-star points (also called axial points).

For each treatment, 10 g of MOF was suspended in 300 mL of deionized water. The suspension pH was adjusted with 1 N NaOH and homogenized for 1 h at 500 rpm on a magnetic stirrer (Guardian 5000; OHAUS, Parsippany, NJ, USA). Ultrasound treatment was applied using an ultrasonicator (VC505; Sonics & Materials, Inc., Newtown, CT, USA) at 30% amplitude with 30 s on/off pulses. The supernatant with the solubilized protein was collected by centrifugation at 3528× *g* for 15 min and filtered through Whatman No. 1 paper. The pH of the filtrate was then adjusted with 2.5 N HCl and allowed to stand for 24 h to promote protein precipitation. The precipitate was recovered by centrifugation at 3528× *g* for 15 min and dehydrated in a drying oven (9053A; Ecoshel, Pharr, TX, USA) at 40 °C for 24 h. Extraction yield and protein content were subsequently determined. The extraction yield was calculated using Equation (3):

(3) $$Extraction\;yield\;(\%)=\frac{TS}{TTS}*100$$

where TS is the total solids obtained after protein extraction (MOPC); TTS is the theoretical total solids and represent the maximum amount of protein powder that could be obtained from *Moringa oleifera* flour, assuming a 100% theoretical recovery.

The protein content of each CCD treatment was determined using the Kjeldahl method [66]. For digestion, 0.7 g of sample, 8.7 g of catalyst mixture, and 10 mL of H_2_SO_4_ were placed in a Kjeldahl digester (DK6; Velp Scientific, Milan, Lom, Italy) and heated at 450 °C for 1 h. Distillation was carried out in a Kjeldahl distiller (UDK 129; Velp Scientific, Milan, Lom, Italy) for 5 min using 35% NaOH. Protein quantification was completed by titration with 0.2 N HCl, and total protein content was calculated using a nitrogen-to-protein conversion factor of 6.25.

### 4.4. Nutritional Composition of MOF and MOPC

The nutritional composition of MOF and MOPC was analyzed using standard AOAC methods. Moisture content was determined gravimetrically by oven drying (AOAC 925.10). Ash content was measured by incineration in a muffle furnace (AOAC 923.03) [56]. Total protein was quantified using the Kjeldahl method (AOAC 979.09), while fat content was determined by Soxhlet extraction (AOAC 920.39) [66]. Total dietary fiber, including soluble and insoluble fractions, was assessed using the enzymatic–gravimetric method (AOAC 985.29) [66]. Carbohydrate content was calculated by difference, obtained by subtracting the percentages of moisture, ash, fat (ether extract), protein, and total dietary fiber from 100%.

Mineral content—including calcium, iron, potassium, magnesium, sodium, and lead—was analyzed in MOF using an atomic absorption spectrometer (AAnalyst 200; PerkinElmer, Inc., Waltham, MA, USA). Amino acid composition of MOF and MOPC was determined by acid hydrolysis, with asparagine and glutamine converted to aspartic acid and glutamic acid, respectively. Tryptophan was quantified separately by alkaline hydrolysis followed by high-performance liquid chromatography (1200 Infinity Series; Agilent Technologies, Santa Clara, CA, USA). All amino acids were then quantified by high-performance liquid chromatography after pre-column derivatization, using o-phthalaldehyde for primary amino acids and 9-fluorenylmethyl chloroformate for secondary amino acids [67].

### 4.5. Physicochemical and Functional Properties of MOPC

The pH of MOPC was measured using a potentiometer (Aquasearcher A-AB23PH; OHAUS, Parsippany-Troy Hills, NJ, USA). For measurement, 3 g of MOPC was suspended in 30 mL of distilled water and stirred for 1 min prior to recording the pH.

Water activity was determined by placing 3 g of MOPC in a 4 cm diameter container and analyzing with a water activity meter (LabSwift-aw; Novasina AG, Lachen, ZH, Switzerland).

Color parameters were measured using a colorimeter (BYK Gardner, Columbia, MD, USA) under standard illuminant D65 and 10° observer conditions at 24 °C. Samples were placed in a 4 cm-diameter container with a thickness of 1 cm, and lightness (L*), chroma (C*), and hue angle (h°) were recorded.

Water absorption capacity and oil absorption capacity were determined following the same procedure [68]. Briefly, 300 mg of MOPC was weighed and homogenized in 4.5 mL of distilled water. The mixture was vortexed for 10 min using a vortex mixer (SC-VTX-5; CScientific, Ciudad de Mexico, Mexico), then mixed for 1 h on a roller shaker (CVP-2000P; CScientific) and centrifuged at 3528× *g* for 10 min. The supernatant was decanted, and water absorption capacity was calculated as the amount of water absorbed per gram of protein. Oil absorption capacity was determined using the same procedure, replacing water with canola oil.

Protein solubility was evaluated by suspending 1 g of MOPC in 100 mL of distilled water, adjusting the pH to 2, 4, 6, 8, or 10 with 2.5 N HCl or 1 N NaOH, and centrifuging at 3528× *g* for 10 min. Soluble protein in the supernatant was quantified using the Bradford method, while total protein content was determined by the Kjeldahl method. Protein solubility was expressed as the percentage of soluble protein relative to total protein [69], according to Equation (4):

(4) $$Solubility\;(\%)=\frac{Protein\;in\;the\;supernatant\;(\%)}{Total\;protein\;(\%)}*100$$

Gelation capacity was determined according to methods described in the literature [70]. MOPC was homogenized in distilled water at a 14:100 (*w*/*v*) ratio, and the pH was adjusted to 2, 4, 6, 8, or 10. Aliquots of 5 mL from each solution were transferred into separate tubes, heated in a water bath at 92 °C for 1 h, and then cooled at 4 °C for 2 h. Gel formation was measured in milliliters, and gelation (%) was calculated using Equation (5):

(5) $$Gelation\;(\%)=\frac{Gelled\;volume}{Total\;volume}*100$$

Foaming capacity (*FC*) and foam stability (*FS*) were determined following previously described methods [71]. Briefly, 1 g of MOPC was suspended in 100 mL of distilled water, and the pH was adjusted to 2, 4, 6, 8, or 10. Aliquots of 30 mL from each solution were blended for 1 min using a blender (Oster, Ciudad de Mexico, Mexico), and the resulting foam volume was measured in a graduated cylinder. FC was calculated according to Equation (6):

(6) $$FC\;\left(\%\right)=\frac{\left(V2\;-\;V1\right)\;}{V2}*100$$

where *V*2 is the volume of the protein solution after mixing and *V*1 is the volume of the solution before mixing.

*FS* was determined by measuring the foam volume after 30 min and calculated according to Equation (7):

(7) $$FS\;(\%)=\frac{Final\;foam\;volume}{Total\;volume\;of\;foam}*100$$

Emulsifying capacity (*EC*) and emulsifying stability (*ES*) were determined according to methods reported in the literature [72]. For *EC*, 1 g of MOPC was suspended in 100 mL of distilled water, and the pH was adjusted to 2, 4, 6, 8, or 10. Then, 10 mL of each solution was combined with 15 mL of canola oil in Falcon tubes. Emulsions were formed by homogenizing the mixtures on an orbital shaker (CVP-2000P; CScientific) and subsequently centrifuged at 3087× *g* for 2 min. *EC* was calculated according to Equation (8):

(8) $$EC\;(\%)=\frac{Emulsifying\;layer\;volume}{Volume\;of\;the\;total\;contents\;of\;the\;tube}$$

*ES* was evaluated by heating the emulsions at 80 °C for 30 min, followed by centrifugation at 3087× *g* for 2 min. *ES* was calculated according to Equation (9):

(9) $$ES\;(\%)=\frac{Final\;volume\;of\;the\;emulsifying\;layer}{Initial\;volume\;of\;the\;emulsifying\;layer}$$

### 4.6. Statistical Analysis

*Moringa oleifera* leaf yield and protein extraction were optimized using a CCD within the framework of RSM, comprising 17 experimental runs, including 3 replicates at the central point and 2-star points. Three factors were evaluated at three levels each: solubilization pH (9, 10, and 11), extraction time (10, 20, and 30 min), and precipitation pH (3.5, 4.5, and 5.5). Statistical analyses were performed using Statgraphics Centurion XVI and JMP v.6 (SAS Institute Inc., Cary, NC, USA). Data were analyzed by Student’s *t*-test or one-way ANOVA followed by Tukey’s multiple comparisons. Differences were considered statistically significant at *p* ≤ 0.05. Results are reported as mean ± SD (*n* = 3).

## Figures and Tables

**Table 1:** CCD for optimization of protein extraction from *M. oleifera* leaves.

Experimental Run	Solubilization pH	Extraction Time (min)	Precipitation pH	Extraction Yield (%)	Protein Content (%)
1	8.31	20	4.5	45.07	42.47
2	9	10	3.5	51.4	50.75
3	11	10	3.5	65.18	50.84
4	10	20	4.5	64.48	52.84
5	11.68	20	4.5	82.44	50.39
6	10	36.81	4.5	75.75	46.45
7	10	20	4.5	65.43	52.17
8	11	30	5.5	65.44	51.69
9	9	30	5.5	52.80	50.92
10	9	10	5.5	45.95	53.52
11	10	20	6.18	52.40	50.40
12	11	30	3.5	78.21	47.71
13	10	3.18	4.5	45.33	55.45
14	11	10	5.5	57.76	51.53
15	10	20	4.5	65.65	51.53
16	10	20	2.81	67.25	49.28
17	9	30	3.5	65.86	45.38

**Table 2:** ANOVA for extraction yield.

Source	Sum of Squares	DF	Mean Square	*F*-Value	*p*-Value
A	942.096	1	942.096	51.63	0.0002
B	635.764	1	635.764	34.84	0.0006
C	296.881	1	296.881	16.27	0.0050
A^2^	5.32921	1	5.32921	0.29	0.6057
A × B	0.045	1	0.045	0.00	0.9618
A × C	0.3528	1	0.3528	0.02	0.8933
B^2^	37.5154	1	37.5154	2.06	0.1947
B × C	20.9952	1	20.9952	1.15	0.3190
C^2^	48.6333	1	48.6333	2.67	0.1466
Residual error	127.721	7	18.2459		
Corrected total	2091.12	16			
R^2^ = 93.89%					
Adjusted R^2^ = 86.03%					
Standard error of estimate = 4.27					
Mean absolute error = 2.10					
Durbin–Watson statistic = 2.59 (*p* = 0.857)					
Lag-1 residual autocorrelation = −0.36					

A = solubilization pH; B = ultrasound time; C = precipitation pH.

**Table 3:** ANOVA for protein content.

Source	Sum of Squares	DF	Mean Square	*F*-Value	*p*-Value
A	15.4375	1	15.4375	3.13	0.1200
B	49.7893	1	49.7893	10.10	0.0155
C	16.177	1	16.177	3.28	0.1129
A^2^	33.3679	1	33.3679	6.77	0.0353
A × B	3.125	1	3.125	0.63	0.4520
A × C	1.6562	1	1.6562	0.34	0.5803
B^2^	0.168812	1	0.168812	0.03	0.8584
B × C	4.59045	1	4.59045	0.93	0.3666
C^2^	2.98783	1	2.98783	0.61	0.4617
Residual error	34.4952	7	4.92789		
Corrected total	160.594	16			
R^2^ = 78.52%					
Adjusted R^2^ = 50.90%					
Standard error of estimate = 2.21					
Mean absolute error = 1.16					
Durbin–Watson statistic = 2.22 (*p* = 0.475)					
Lag-1 residual autocorrelation = −0.32					

A = solubilization pH; B = ultrasound time; C = precipitation pH.

**Table 4:** Proximate composition of MOF and MOPC.

Component (g/100 g)	MOF	MOPC
Water	7.71 ± 0.30 ^a^	5.09 ± 0.26 ^b^
Protein	29.38 ± 1.00 ^b^	53.97 ± 1.43 ^a^
Lipids	5.46 ± 0.38 ^b^	15.35 ± 0.15 ^a^
Carbohydrates	10.44 ± 0.62 ^a^	5.43 ± 0.56 ^b^
Dietary fiber	37.98 ± 1.16 ^a^	18.09 ± 0.64 ^b^
Insoluble fiber	32.06 ± 1.88 ^a^	17.38 ± 0.99 ^b^
Soluble fiber	5.92 ± 0.89 ^a^	0.71 ± 0.07 ^b^
Ash	9.03 ± 0.05 ^a^	2.07 ± 0.041 ^b^

Data are expressed as mean ± standard deviation of triplicate measurements. Different letters in the same row denote statistically significant differences at *p* < 0.05, as determined by Student’s *t*-test.

**Table 5:** Mineral content in MOF.

Mineral	MOF (mg/100 g)	RDDI (1–3 Years) (mg/Day)	RDDI (19–30 Years) (mg/Day)
Calcium	1751.85 ± 3.60	500	1000
Magnesium	512.55 ± 1.80	80	100–310
Potassium	1144.58 ± 1.06	3000	4700
Iron	8.73 ± 0.05	5.8	13.7–29.4
Sodium	85.81 ± 1.06	1000	1500
Lead	ND	-	-

RDDI = recommended daily dietary intake according to Pan American Health Organization (2020) [39].

**Table 6:** Amino acid profile of the protein present in MOF and MOPC.

Amino Acids	FAO and IAEA (2024) [42] (g/kg/d of Protein)	MOF (g/kg of Protein)	MOPC (g/kg of Protein)
Histidine	15	18.18	16.08
Isoleucine	30	50.29	48.64
Leucine	59	10.44	110.98
Lysine	45	102.13	94.90
Methionine + cystine	22	26.31	13.30
Phenylalanine + tyrosine	38	78.53	86.76
Threonine	23	54.16	42.29
Tryptophan	6	11.61	38.71
Valine	30	63.83	57.18
Arginine	-	59.19	63.93
Aspartic acid	-	70.79	63.33
Glycine	-	112.57	100.66
Glutamic acid	-	50.68	43.88
Serine	-	229.01	136.39
Proline	-	41.01	33.35

**Table 7:** Physicochemical and functional properties of MOPC obtained by ultrasound.

Parameters					
pH	4.63 ± 0.01				
Water activity (a_w_)	0.44 ± 0.007				
Color					
L* (lightness)	23.90 ± 1.57				
C* (chroma)	16.36 ± 0.58				
h° (hue angle)	88.11 ± 1.09				
Water absorption (g/g)	1.66 ± 0.07				
Oil absorption (g/g)	2.52 ± 0.18				
	pH 2	pH 4	pH 6	pH 8	pH 10
Solubility (%)	24.26 ± 0.15 ^a^	0.23 ± 0.05 ^e^	6.54 ± 0.56 ^d^	21.65 ± 0.12 ^c^	22.42 ± 0.15 ^b^
Gelling (%)	55.55 ± 9.62 ^b^	28.88 ± 7.69 ^b,c^	55.55 ± 19.24 ^b^	100 ± 0.00 ^a^	6.66 ± 0.00 ^c^
Foaming capacity (%)	21.11 ± 1.92 ^a^	15.55 ± 3.84 ^a^	3.33 ± 0.00 ^b^	15.00 ± 1.66 ^a^	20.00 ± 3.33 ^a^
Foam stability (%)	94.44 ± 9.62 ^a^	41.66 ± 14.43 ^b^	30.10 ± 26.27 ^b^	41.38 ± 23.57 ^b^	35.95 ± 9.59 ^b^
Emulsifying capacity (%)	27.60 ± 6.60 ^b^	4.00 ± 0.00 ^c^	4.00 ± 0.00 ^c^	0.00 ± 0.00 ^c^	58.66 ± 2.30 ^a^
Emulsion stability (%)	14.78 ± 5.52 ^c^	36.66 ± 11.54 ^b^	36.66 ± 11.54 ^b^	0.00 ± 0.00 ^c^	79.52 ± 0.82 ^a^

Data are presented as mean ± standard deviation of triplicate measurements. Different letters in the same row indicate statistically significant differences at *p* < 0.05, according to one-way ANOVA followed by Tukey’s test.

**Table 8:** Central Composite Desing under the Response Surface Methodology framework.

Experimental Run	Solubilization pH	Extraction Time (minutes)	Precipitation pH
1	8.31	20	4.5
2	9	10	3.5
3	11	10	3.5
4	10	20	4.5
5	11.68	20	4.5
6	10	36.81	4.5
7	10	20	4.5
8	11	30	5.5
9	9	30	5.5
10	9	10	5.5
11	10	20	6.18
12	11	30	3.5
13	10	3.18	4.5
14	11	10	5.5
15	10	20	4.5
16	10	20	2.81
17	9	30	3.5

## Data Availability

The original contributions presented in this study are included in the article. Further inquiries can be directed to the corresponding author.
